# Self-reported oral health-related quality of life and caries experiences of 5-year-old children in Mandalay, Myanmar

**DOI:** 10.1186/s12903-023-03803-4

**Published:** 2024-01-06

**Authors:** Saw Nay Min, Duangporn Duangthip, Sherry Shiqian Gao, Palinee Detsomboonrat

**Affiliations:** 1 Department of Community Dentistry, Faculty of Dentistry, Chulalongkorn University, Bangkok, Thailand; 2 Restorative Dental Sciences, Faculty of Dentistry, The University of Hong Kong (HKU), Pokfulam, Hong Kong; 3 Department of Stomatology, School of Medicine, Xiamen University, Xiamen, China

**Keywords:** Children, Dental caries, Myanmar, Oral health-related quality of life, SOHO-5

## Abstract

**Background:**

This study aimed to examine the impact of dental caries and other potential socio-demographic factors on the oral health-related quality of life (OHRQoL) of preschool children from Myanmar. This was done using the Scale of Oral Health Outcomes for 5-year-old children (SOHO-5) as reported by both the children and their parents.

**Methods:**

A structured questionnaire was conducted to collect demographic information about the children and their caregivers, as well as socioeconomic data. The OHRQoL was assessed by interviewing the children and their parents using the Myanmar versions of SOHO-5c and SOHO-5p, respectively. Caries experience was assessed by two calibrated examiners and recorded using the dmft index. The Poisson regression model was adopted to investigate the association between OHRQoL and dental caries including socioeconomic factors.

**Results:**

A total of 454 pairs participated in the study. Among them, 64% of children and 70% of parents reported a negative impact on OHRQoL (with SOHO-5c and SOHO-5p scores exceeding 0). The mean score (SD) of the child self-report and parental version of the SOHO-5 was 1.86 (2.27) and 2.65 (3.13), respectively. Difficulties in eating and sleeping were the most commonly reported by both children and parents. The overall prevalence of dental caries was 87% (mean dmft score:5.59, SD:4.65). The final multivariate-adjusted model revealed that children with higher caries experiences were more likely to have lower OHRQoL for both child self-report (RR 4.38, 95% CI 3.16–6.14, *p* < 0.001) and parental report (RR 6.07, 95% CI 4.38–8.41, *p* < 0.001), respectively. A lower family income had a negative impact on the children’s OHRQoL in child self-report (RR 1.59, 95% CI 1.26–2.04, *p* < 0.001) and parental report (RR 1.46, 95% CI 1.19–1.78, *p* < 0.001).

**Conclusion:**

Two-thirds of the study children and their parents perceived the negative impact on children’s OHRQoL. Higher caries experience and lower family income were associated with poorer OHRQoL of 5-year-old Myanmar children.

**Supplementary Information:**

The online version contains supplementary material available at 10.1186/s12903-023-03803-4.

## Background

Dental caries is one of the most frequent chronic oral diseases in children globally [1]; if left untreated, it can result in pain, abscess, and both local and systemic infections [2]. Untreated caries may give rise to consequences that detrimentally affect the quality of life in children [3] such as impairment of chewing and speaking abilities, diminished school performance, psychological disturbances, e.g., disturb in sleeping, and factors related to social relations, such as avoid smiling and speaking [4, 5]. Furthermore, a negative impact on the family, e.g., parents’ guilt, work absenteeism, and financial difficulties was reported among the children with untreated caries experiences [6].

Despite having decreased globally, dental caries remains prevalent in certain developing countries [7, 8]. The Republic of the Union of Myanmar, a country whose economic development is ongoing, was estimated to contain 51.4 million people in 2014 according to Myanmar Population Census records. Of this population, 30% live in urban areas, while the remaining 70% reside in rural areas [9]. In spite of the introduction of a National Health Plan (2017–2021) aimed at achieving Universal Health Coverage (UHC), oral health has been given limited emphasis in the majority of healthcare strategies [10, 11]. Due to the limited ratio of dentists to the population in Myanmar, carrying out oral health education programs and the provision of oral health services throughout the country is a challenge [12]. Moreover, although evidence-based programs prevention and management of dental caries have been implemented in certain locations [13], caries prevalence in school children remains high, which can be attributed to the lack of knowledge of oral health, the limited supply of restorative material and personnel [10, 14].

According to the National Oral Health survey (2017), the mean dmft of 6-year-old-school children was 5.7 whereas the prevalence of untreated caries was 84.1%. However, there is no information about the effect of dental caries on the oral health-related quality of life (OHRQoL) among young Myanmar children. Even though parental proxy measures are typically used to assess the OHRQoL of young children [15, 16], there’s a counterargument stating that a child’s actual oral health status can be effectively represented solely through parent proxy reports [17]. In recent times, the Scale of Oral Health Outcomes for 5-year-old children (SOHO-5) was developed to assess the impact of dental caries on OHRQoL of young children using both the children and parental reports [18]. The findings of recent research established the Myanmar SOHO-5 version to have a high degree of reliability and validity in a 5-year-old Myanmar population [19]. Oral health information about Myanmar preschool children is scarce [20] and updated information about the impact of dental caries on the OHRQoL of preschool children would be beneficial for the development of effective oral health preventive programmes to improve the oral health status of the children population [21].

The aim of the study was to assess the impact of dental caries and other potential factors on oral health related quality of life among 5-year-old children in Mandalay, in addition to analyzing the agreement level between parent-child pairs.

## Methods

This cross-sectional study was conducted from December 2021 to February 2022 in Mandalay, Myanmar. Ethical approval was received from the Human Research Ethics Committee (HREC-DCU 2021-047) at Chulalongkorn University.

### Population and sample size calculation

The study population comprised 5-year-old children and their parents living in Mandalay City. Mandalay City is the middle part of Myanmar and the second-largest city which consists of seven subdistricts. According to the Myanmar Information Management Unit (MIMU) 2019, the total number of 5-year-old school children was 21,160 in seven districts. The calculation of the sample size was performed by Poisson regression using the G*Power software to achieve a statistical power of 95% (to minimize Type II error), employing a two-sided test with a significance threshold of 0.05. The smallest detectable rate ratio (RR) for the OHRQoL was set at 1.4 when comparing children without caries (unexposed) to those with caries experience (exposed) [22]. Thus, a total of 362 participants were determined to be necessary. Considering an estimated participation rate of 80%, it was determined that at least 452 participants would need to be invited to participate in the study. This study employed a quota sampling technique, which involved selecting participants in proportion to the child population in each district (MIMU, 2019) [23]. Five-year-old children from 14 schools across 7 districts were recruited for the study. Informed consent was acquired from the parent or legal guardian of the participants prior to the implementation. The inclusion criteria were children who were 5 years old, in good health, proficient in speaking Burmese, and whose parents possessed an understanding of the Burmese language. Children with disabilities, developmental delays, uncooperative behavior, or those who refused to undergo the oral examination were excluded from the study.

### Questionnaire survey

Regarding the child’s OHRQoL, the parent and child were independently interviewed face-to-face using the Myanmar SOHO-5 questionnaire. The SOHO-5 questionnaire is composed of a child self-report and a parental report for the history of the child’s oral health [18]. Both reports were comprised of seven items, six of which were similar in terms of content. Scores on a three-point scale ranging from 0 to 14 were attributed to the responses given by the child report while a five-point scale of 0 to 28 was assigned to the parental report (Appendix). For both SOHO-5c and SOHO-5p, a higher score refers to a greater negative impact on the OHRQoL of children.

Furthermore, the parent’s educational background and family income were also interviewed. The family income was determined by utilizing the Myanmar minimum wage of 4800 Kyat per day or 150,000 Kyat monthly in 2021 as a baseline. The participants underwent interviews conducted by three trained interviewers on the same day before the clinical examinations were performed.

### Children’s oral examination

An oral examination was performed while the child was in a seated position on a chair under natural light. The caries status of the children was assessed by two calibrated examiners according to World Health Organization criteria (WHO) [24] using a penlight, disposable dental mirror, and WHO-CPI probe. Training and calibration exercises were conducted on volunteers (35 children) before the study. Caries experience in primary teeth was recorded in a modified oral health assessment form using dmft index. For the recording of the teeth, the following criteria were used: decayed (dt): an unmistakable dentine carious cavity or filled lesion with recurrent caries, missing (mt): extracted due to caries, filled (ft): a permanent filling without caries. The participants’ dmft score was divided into three groups, based on the scoring procedure of a previous study [25]: (1) dmft 0 (no caries experiences), (2) dmft 1–5 and (3) dmft ≥ 6.

### Data analysis

Data were analyzed by using the SPSS version 22.00 software (IBM Corp). The statistical analyses encompassed calculations of means, frequencies, and percentages. These analyses were conducted to assess the caries experiences, OHRQoL among children, and the socio-demographic status of the parents. Cohen’s kappa coefficient was used to evaluate the intra- and inter-examiner reliability for caries diagnosis.

Poisson regression analysis with robust variance was constructed to correlate the total SOHO-5 scores to caries experiences and sociodemographic factors such as the child’s gender, parent’s education and family income. In these analyses, the outcome was treated as a count variable, consistent with the approach adopted in prior studies [22, 25], and we computed rate ratios (RR) along with corresponding 95% confidence intervals (95% CI). All variables were incorporated into the final multivariate model; nevertheless, variables that exhibited non-significance (*p* > 0.05) were subsequently excluded from the final model. The parent-child agreement for six common items was evaluated by analyzing the intra-class correlation coefficient (ICC) between the total and item scores of the child’s and parents’ reports [26]. The agreement level determined by the ICC was categorized as poor (< 0.20), weak (0.20–0.40), moderate (0.41–0.60), substantial (0.61–0.80), and excellent (0.81-1.0) [25, 27].

## Results

A total of 509 child-parent pairs were invited to participate in the study, out of which 489 had received written consent prior to the implementation of the study, rendering a response rate of 96%. Twenty-nine children were absent, and six children refused the oral examination on the day of the examination, resulting in 454 children being enrolled in the study. There was a strong consensus in the assessment of caries, indicated by a kappa value (k) of 0.87, which reflects high inter-examiner reliability. Furthermore, when around 10% of the participants underwent a second evaluation two weeks later, the intra-examiner reliability was found to be outstanding, with a kappa value of 0.90.

Table 1 revealed the caries status and sociodemographic characteristics of the participants. In total, 87% of the subjects had caries experiences (dmft > 0), with a mean dmft index of 5.59 (with a standard deviation of 4.65). The majority of respondents for the parent version were mothers (87.7%). Table 2 showed the distribution of responses to each item in SOHO-5 reports of both children and parents. Overall, 64.1% of children experienced an impact on oral health (SOHO-5 > 0), and this finding was corroborated by parental reports of 70.5%. The highest reported score on the child self-report version was 11 out of 14, while the highest score on the parental version was 20 out of 28. The mean total score of the SOHO-5 was 1.86 ± 2.27 for the child self-report and 2.65 ± 3.13 for the parental report. Of the items analyzed within the SOHO-5, both reports demonstrated that eating difficulties represented the highest impact item on OHRQoL, followed by difficulty in sleeping.

Unadjusted and adjusted associations between caries experience, sociodemographic variables, and mean total SOHO-5 scores were shown in Tables 3 and 4. Both reports revealed that an increased SOHO-5 score (*p* < 0.05) was associated with higher caries experiences and lower family incomes. In comparison to children without caries experiences, those with moderate caries experiences exhibited a SOHO-5 score 2.24 times higher (RR 2.24, 95% CI 1.58–3.18, *p*-value < 0.005), while those with high caries experiences had a score 4.38 times higher (RR 4.38, 95% CI 3.13–6.14, *p*-value < 0.001). Similarly, in terms of the parental report, the corresponding values were RR 3.13 (95% CI 2.24–4.36, *p*-value < 0.001) for moderate caries experiences and RR 6.07 (95% CI 4.38–8.41, *p*-value < 0.001) for high caries experiences. A decrease in family income exhibited a negative impact on the children’s OHRQoL in both child self-report (RR 1.58, 95% CI 1.26–1.99, *p* < 0.001) and parental report (RR 1.49, 95% CI 1.24–1.80, *p* < 0.001). The parent-child pairs indicated an ICC of 0.78 (95% CI: 0.75–0.82) on the total score and had item ICC from 0.28 to 0.70 (Table 5).

**Table 1:** Characteristics of children and parents

Characteristics	Frequency	Percentage
Sex		
Female Male	234 220	51.5 48.5
Parent interviewed		
Mother Father	398 56	87.7 12.3
Mother’s education		
Junior secondary school Secondary school University or Post-secondary school	120 168 166	26.4 37.0 36.6
Father’s education		
Junior secondary school Secondary school University or Post-secondary school	113 159 182	24.9 35.0 40.1
Monthly household income in Kyat ($)		
> 300,000 ($162.01) 150,000–300,000 ($ 81.01–162.01) < 150,000 ($ 81.01)	156 260 38	34.4 57.3 8.4
Caries experience (dmft)		
Caries free (dmft = 0) Moderate caries experience (dmft 1–5) High caries experience (dmft ≥ 6)	59 186 209	13.0 41.0 46.0

**Table 2:** Distribution of SOHO-5 responses to Children’s and parent’s reports

Child self-report						
Item	No n (%)	A little n (%)	A lot n (%)	Mean (SD)		
Difficulty in eating	180 (39.6)	176 (38.8)	98 (21.6)	0.82 (0.76)		
Difficulty in drinking	404 (89.0)	49 (10.8)	1 (0.2)	0.11 (0.32)		
Difficulty in speaking	404 (89.0)	44 (9.7)	6 (1.3)	0.12 (0.36)		
Difficulty in playing	417 (91.9)	34 (7.5)	3 (0.7)	0.09 (0.30)		
Difficulty in sleeping	289 (63.7)	131 (28.9)	34 (7.5)	0.44 (0.63)		
Avoid smiling due to pain	383 (84.4)	64 (14.1)	7 (1.5)	0.17 (0.41)		
Avoid smiling due to appearance	407 (89.6)	46 (10.1)	1 (0.2)	0.11 (0.31)		
Total score				1.86 (2.27)		
Parent’s report						
Item	Not at all (%)	A little (%)	Moderate (%)	A lot (%)	A great deal (%)	Mean (SD)
Difficulty in eating	185 (40.7)	138 (30.4)	78 (17.2)	45 (9.9)	8 (1.8)	1.02 (1.06)
Difficulty in speaking	384 (84.6)	54 (11.9)	14 (3.1)	2 (0.4)	0	0.19 (0.49)
Difficulty in playing	405 (89.2)	39 (8.6)	7 (1.5)	2 (0.4)	1 (0.2)	0.14 (0.45)
Difficulty in sleeping	271 (59.7)	120 (26.4)	48 (10.6)	14 (3.1)	1 (0.2)	0.58 (0.81)
Avoid smiling due to pain	371 (81.7)	64 (14.1)	14 (3.1)	5 (1.1)	0	0.24 (0.55)
Avoid smiling due to appearance	408 (89.9)	38 (8.4)	6 (1.3)	2 (0.4)	0	0.12 (0.40)
Influence self-confidence	342 (75.3)	81 (17.8)	18 (4.0)	9 (2.0)	4 (0.9)	0.35 (0.73)
Total score						2.65 (3.12)

**Table 3:** Association between caries experience and sociodemographic factors with SOHO-5 score for child self-report

Covariates	Unadjusted model			Adjusted model	
	Robust RR (95% CI)	*p*-value		Robust RR (95% CI)	*p*-value
**Caries experiences**					
Caries free* Moderate caries experiences (1–5) High caries experiences ( ≥ 6)	2.28 (1.61–3.23) 4.59 (3.28–6.43)	< 0.005 < 0.001		2.24 (1.58–3.18) 4.38 (3.13–6.14)	< 0.005 < 0.001
**Child gender**					
Female* Male	0.94 (0.75–1.18)	0.593		CNS	
**Mother’s education**					
Junior secondary school* Secondary school University or Post-secondary school	1.05 (0.79–1.40) 1.01 (0.75–1.34)	0.721 0.981		CNS	
**Father’s education**					
Junior secondary school* Secondary school University or Post-secondary school	0.89 (0.67–1.19) 0.92 (0.70–1.22)	0.450 0.580		CNS	
**Monthly household income (MMK)**					
> 300,000* 150,000–300,000 < 150,000	1.49 (1.28–1.75) 1.87 (1.48–2.35)	< 0.001 < 0.001		1.39 (1.19–1.62) 1.58 (1.26–1.99)	< 0.001 < 0.001

*Reference; Robust RR: Robust rate ratio; CNS: covariate not selected for final model (p > 0.05)

**Table 4:** Association between caries experience and sociodemographic factors with SOHO-5 score for Parent report

Covariates	Unadjusted model		Adjusted model	
	Robust RR (95% CI)	*p*-value	Robust RR (95% CI)	*p*-value
**Caries experiences**				
Caries free* Moderate caries experiences (1–5) High caries experiences ( ≥ 6)	3.16 (2.27–4.41) 6.30 (4.55–8.72)	< 0.001 < 0.001	3.13 (2.24–4.36) 6.07 (4.38–8.41)	< 0.001 < 0.001
**Child gender**				
Female*			CNS	
Male	0.92 (0.74–1.15)	0.480		
**Mother’s education**				
Junior secondary school*				
Secondary school University or Post-secondary school	1.07 (0.81–1.42) 1.05 (0.80–1.40)	0.623 0.688	CNS	
**Father’s education**				
Junior secondary school*				
Secondary school University or Post-secondary school	0.96 (0.72–1.28) 1.01 (0.77–1.33)	0.781 0.935	CNS	
**Monthly household income (MMK)**				
> 300,000*				
150,000–300,000 < 150,000	1.30 (1.15–1.48) 1.77 (1.47–2.13)	< 0.001 < 0.001	1.21 (1.06–1.36) 1.49 (1.24–1.80)	< 0.005 < 0.001

*Reference; Robust RR: Robust rate ratio; CNS: covariate not selected for final model (p > 0.05)

**Table 5:** Correlations between parent-child pairs for SOHO-5 total and items score

Item	Parent-child ICC (95% CI)	*p*-value
Total score	0.78 (0.75–0.82)	< 0.001
Difficulty in eating	0.70 (0.65-0.74)	< 0.001
Difficulty in speaking	0.52 (0.45–0.58)	< 0.001
Difficulty in playing	0.49 (0.42–0.56)	< 0.001
Difficulty in sleeping	0.66 (0.61–0.71)	< 0.001
Avoid smiling due to pain	0.62 (0.56–0.68)	< 0.001
Avoid smiling due to appearance	0.28 (0.20–0.37)	< 0.001

ICC: Intraclass correlation coefficient

## Discussion

This is the first study reporting the association between early childhood caries and OHRQoL reported by children and their parents in Myanmar where the prevalence of untreated ECC is very high. The findings of this study highlighted a significant association between dental caries and OHRQoL among five-year-old children, as perceived by both the children themselves and their parents. Caries prevalence and dmft value in children were very high among the participants. The items ‘difficulty eating’ and ‘difficulty in sleeping due to pain’ had the highest mean scores reported by both children and parents, which is in agreement with the previous studies [25, 28, 29]. Overall, caries was found to be the primary oral clinical condition responsible for impacting all aspects assessed in the study. Children with higher caries experience have more chances of having a negative impact on their OHRQoL in this study. This result is similar to the previous studies in Hong Kong [30], Trinidad [3] and Brazil [31].

Studies conducted previously have demonstrated that parents’ education level and socio-economic status are risk factors that impact a child’s oral health and OHRQoL [21, 32]. The Poisson analysis of caries experiences and socio-demographic factors in our model revealed that a family income exceeding 300,000 Kyat had a positive impact on the OHRQoL of young children; this result is similar to that which was previously reported [25]. Children with low family income may be more likely to have poorer OHRQoL due to a lack of access to regular dental care, limited resources for preventive care, and a lack of prioritization for children’s oral health needs. It is necessary to target these groups of children by improving access to dental care to relieve pain and infection as well as improving the parent’s oral health knowledge and encouraging positive oral health behaviors with parental involvement.

Initiating preventive measures and fundamental treatments in early childhood is crucial, as this period offers the most cost-efficient opportunity for intervention [33]. This approach should include considering sources of dietary fluoride, such as fluoridated water or milk. Moreover, it’s important to implement school health programs that establish consistent toothbrushing practices and encourage healthy dietary choices. These strategies are vital in managing Early Childhood Caries (ECC) in young children [34]. Additionally, the use of silver diamine fluoride has recently emerged as a notable, non-invasive, and economical option for treating caries [35]. This treatment is particularly beneficial in reaching children in remote or underserved regions, thereby enhancing dental care accessibility. This is especially relevant for children and families living in poverty in Myanmar, where there is a high prevalence of ECC and a general lack of quality dental care. Further work is required to investigate the impact of alternative caries management on the improvement of OHRQoL in children in Myanmar.

Approximately two-thirds of the children and the parents demonstrated an adverse effect on OHRQoL (SOHO-5 score > 0) of the children for at least one item. This finding also showed that children’s self-reports can be reliable for their oral health information and the perceptions of parents compared with the children’s perception allow a more comprehensive evaluation of the child’s OHRQoL [29]. Despite the high caries prevalence in this study, the mean total SOHO-5 scores of both reports demonstrated low overall scores on OHRQoL of the children. This finding suggests that the primary concerns in Myanmar were disturbances in eating and sleeping functions. Differences in cultural beliefs and societal values related to child oral health may play a role in this aspect.

It is important to acknowledge the limitations of this study. The present study is a cross-sectional design and therefore, there was no evidence for causality between dental caries and OHRQoL of children and we cautiously generalize from the study sample to other populations. Therefore, the results could not be generalized to all Myanmar children. However, the acceptable participation rate, sufficient sample size, use of a validated instrument, and good inter and intra-examiner reliability assure the findings of this study. Nevertheless, the present findings could provide crucial information on the association of dental caries status and OHRQoL of Myanmar 5-year-old children for the public health policymakers regarding the future development of oral health promotion programs for preschool children. Future longitudinal studies are recommended to investigate the causal effect of dental caries on OHRQoL of the children and their parents using a presentative sample.

## Conclusion

Two-thirds of both the children and their parents perceived a negative impact on the children’s OHRQoL. Children with higher caries experience and lower family income are likely to have poorer OHRQoL. It is recommended to promote collaboration among healthcare professionals, dental associations, community organizations, and government bodies to develop a comprehensive approach aimed at enhancing the quality of life of young children.

### Electronic supplementary material

Below is the link to the electronic supplementary material.

Supplementary Material 1

## Data Availability

The datasets used and/or analyzed during the current study are not publicly available due to the data protection guidelines according to the ethics approval but are available from the corresponding author on reasonable request.

## Abbreviations

UHC: Universal Health Coverage
OHRQoL: Oral health-related quality of life
SOHO-5: Scale of Oral Health Outcomes for 5-year-old children
MIMU: Myanmar Information Management Unit
RR: Rate ratio
SOHO-5c: Scale of Oral Health Outcomes for 5-year-old children for child self-report
SOHO-5p: Scale of Oral Health Outcomes for 5-year-old children for parent self-report
WHO: The World Health Organization
CPI: Community periodontal index
dmft: Decayed, Missing, and Filled Teeth Index for primary teeth
dt: Decayed Teeth
mt: Missing Teeth
ft: Filled Teeth
CI: Confidence intervals
ICC: Intra-class correlation coefficient
MMK: Myanmar Kyat
ECC: Early childhood caries
SDF: Silver diamine fluoride
