# Supplementary material for: Self-reported oral health-related quality of life and caries experiences of 5-year-old children in Mandalay, Myanmar
Source: BMC Oral Health. 2024 Jan 6;24:31. doi: 10.1186/s12903-023-03803-4 (PMC10771686; doi:10.1186/s12903-023-03803-4)
Supplement: Supplementary file 1 — Supplementary Material 1 [file 12903_2023_3803_MOESM1_ESM.docx]

**Appendix- Scale of Oral Health Outcomes for 5-year-old children (SOHO-5) questionnaires (child and parental version)**

**SOHO-5 QUESTIONNAIRES (Child version)**

No A little A lot

1. Difficulty in eating   

2. Difficulty in drinking   

3. Difficulty in speaking   

4. Difficulty in playing   

5. Avoid smiling due to pain   

6. Avoid smiling due to appearance   

7. Difficulty in sleeping   

**SOHO-5 QUESTIONNAIRES (Parental version)**

Not at all A little Moderate Often Very often

1. Difficulty in eating     

2. Difficulty in speaking     

3. Difficulty in playing     

4. Avoid smiling due to pain     

5. Avoid smiling due to appearance     

6. Difficulty in sleeping     

7. Effect on self esteem     
